# From Indoor to Daylight Electroluminescence Imaging for PV Module Diagnostics: A Comprehensive Review of Techniques, Challenges, and AI-Driven Advancements

**DOI:** 10.3390/mi16040437

**Published:** 2025-04-04

**Authors:** Rodrigo del Prado Santamaría, Mahmoud Dhimish, Gisele Alves dos Reis Benatto, Thøger Kari, Peter B. Poulsen, Sergiu V. Spataru

**Affiliations:** Department of Electrical and Photonics Engineering, Technical University of Denmark, 4000 Roskilde, Sjælland, Denmark; rdpsa@dtu.dk (R.d.P.S.); garb@dtu.dk (G.A.d.R.B.); thkje@dtu.dk (T.K.); ppou@dtu.dk (P.B.P.); sersp@dtu.dk (S.V.S.)

**Keywords:** electroluminescence imaging, photovoltaic diagnostics, outdoor and daylight EL imaging, AI-driven PV defect detection

## Abstract

This review paper presents a comprehensive analysis of electroluminescence (EL) imaging techniques for photovoltaic (PV) module diagnostics, focusing on advancements from conventional indoor imaging to outdoor and daylight EL imaging. It examines key challenges, including ambient light interference and environmental variability, and highlights innovations such as infrared-sensitive indium gallium arsenide (InGaAs) cameras, optical filtering, and periodic current modulation to enhance defect detection. The review also explores the role of artificial intelligence (AI)-driven methodologies, including deep learning and generative adversarial networks (GANs), in automating defect classification and performance assessment. Additionally, the emergence of drone-based EL imaging has facilitated large-scale PV inspections with improved efficiency. By synthesizing recent advancements, this paper underscores the critical role of EL imaging in ensuring PV module reliability, optimizing performance, and supporting the long-term sustainability of solar energy systems.

## 1. Introduction

### 1.1. Overview of Electroluminescence (EL) Imaging

Electroluminescence (EL) imaging is a powerful diagnostic tool widely utilized in the field of photovoltaics (PV) for assessing the health and performance of solar cells and modules. By applying an electrical current to a PV device, EL imaging captures the emitted infrared light using a specialized camera, enabling the identification of defects, cracks, and degradation patterns that are otherwise invisible to the naked eye. This non-invasive method provides high-spatial resolution insights into the structural integrity and operational efficiency of PV systems, making it indispensable for quality control, research, and field inspections [1,2].

Conventionally, EL imaging has been performed in controlled indoor environments to ensure optimal lighting conditions and minimize external interference. However, with the growing deployment of large-scale PV installations, the need for outdoor imaging techniques has become increasingly critical. Outdoor EL imaging, particularly in daylight conditions, presents unique challenges, such as managing ambient light and ensuring sufficient signal-to-noise (SNR) ratios, which have driven significant innovations in this field [3,4,5].

The versatility of EL imaging extends beyond defect detection; it plays a key role in studying degradation mechanisms, predicting long-term performance, and guiding maintenance strategies. Recent advancements, particularly in outdoor in situ EL imaging, have expanded its applications and relevance. As the field continues to evolve, integrating artificial intelligence (AI) for image data analysis can revolutionize how EL imaging is conducted and utilized.

### 1.2. Importance of EL Imaging in Photovoltaic Systems

In solar PV manufacturing, since its introduction as a characterization technique for solar cells [6], EL imaging plays a pivotal role in quality assurance by enabling the detection of microcracks, shunting and soldering defects, and cell-level anomalies during the production process [7,8,9]. These defects, if undetected, can significantly impair the electrical performance and lifespan of solar modules [10,11,12,13,14,15]. An EL signal is created by injecting the current into the PV cell or module; this current creates an excess in electron–hole pairs that produce radiative recombination. By using a camera sensor, one can obtain an image of the EL signal coming from the PV device. Equation (1) defines the relationship between the EL signal intensity ($\phi_{i}$) captured by a camera sensor in each pixel *i* and its exponential relationship with the cell diode voltage ($V_{i}$), where $C_{i}$ is the camera calibration constant and $V_{T}$ is the thermal voltage [16].

(1) $$\phi_{i}=C_{i}\cdot \exp(\frac{V_{i}}{V_{T}})$$

**Figure 1:**
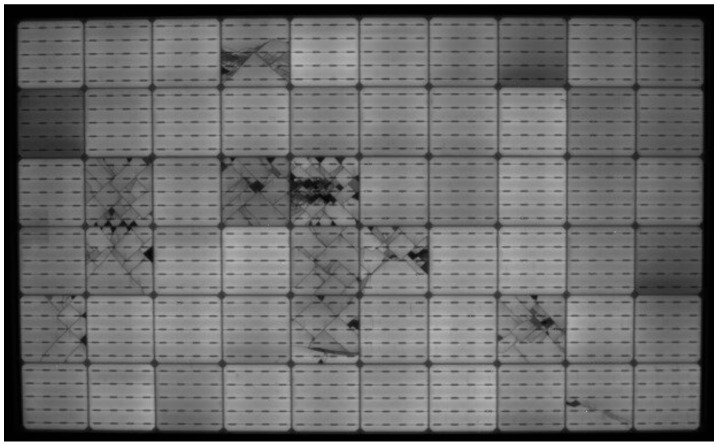
EL image of a PV module showcasing cell cracks and dark cells with shunting, suggesting potential-induced degradation.

By integrating EL imaging into production lines, manufacturers can ensure higher yield rates and improved product reliability, thereby reducing warranty claims and operational risks. Figure 1 illustrates an EL image of a PV module, showcasing various defects such as microcracks, which are critical indicators of degradation. Such diagnostic capabilities make EL imaging a cornerstone in advancing the durability and efficiency of photovoltaic systems.

Beyond the factory floor, EL imaging is instrumental in field inspections for installed PV systems. Over time, solar modules are subjected to various stressors, including thermal cycling, mechanical loading, and environmental exposure, which can lead to cracks, corrosion, and potential-induced degradation (PID) [7,17,18]. EL imaging allows operators to assess the health of PV modules in a non-destructive way, without dismantling them, providing a cost-effective solution for preventive maintenance and fault diagnosis [19]. This capability is particularly valuable for large-scale solar farms, where manual inspections would be time-consuming and impractical.

One of the most significant contributions of EL imaging is its ability to study and understand degradation mechanisms in PV modules. By comparing EL images taken over time, researchers can monitor the progression of defects and correlate them with environmental and operational factors [20,21,22,23,24]. This longitudinal analysis is crucial for improving material selection, module design, and operational strategies, ultimately enhancing the durability and performance of PV systems.

EL imaging also plays a vital role in performance prediction and reliability assessment. By quantifying the severity and distribution of defects, it is possible to estimate the impact on power output and energy yield [25,26,27,28,29,30,31,32,33]. This information not only aids in making informed decisions about repair or replacement but also contributes to financial modeling for energy production and investment planning.

In the research domain, EL imaging serves as a powerful tool for testing novel materials, module architectures, and manufacturing processes [34,35,36,37,38]. It provides high-spatial resolution images that are critical for validating theoretical models and developing next-generation PV technologies. Furthermore, advancements in EL imaging, such as outdoor and real-time imaging techniques, are expanding the boundaries of its applications, making it an indispensable asset for researchers and industry professionals alike.

EL imaging is also aligned with global sustainability goals, particularly as the world transitions to renewable energy. By enabling the early detection of issues and improving the reliability of PV systems, EL imaging supports the long-term viability of solar energy as a cornerstone of the energy transition. Its applications in refurbishing and recycling end-of-life modules further underscore its importance in advancing circular economy principles in the solar industry [39].

### 1.3. Scope and Objectives of the Review

This review aims to provide a comprehensive exploration of the evolution and advancements in EL imaging techniques, with a specific focus on their applications in the PV industry. Starting with the foundational principles and conventional methods of EL imaging, the review examines the transition from controlled indoor environments to challenging outdoor settings, including innovations that have enabled EL imaging under daylight conditions. It highlights key contributions, such as the groundbreaking developments in outdoor EL imaging, which address critical challenges like ambient light interference and operational efficiency.

Furthermore, the review delves into the transformative potential of AI in EL imaging, showcasing how machine and deep learning and data analytics are reshaping defect detection, performance prediction, and operational scalability. By consolidating insights from the existing literature and exploring emerging trends, this review seeks to serve as a valuable resource for researchers, industry practitioners, and stakeholders, offering both a historical perspective and a forward-looking vision for the integration of EL imaging into next-generation PV systems.

**Figure 2:**
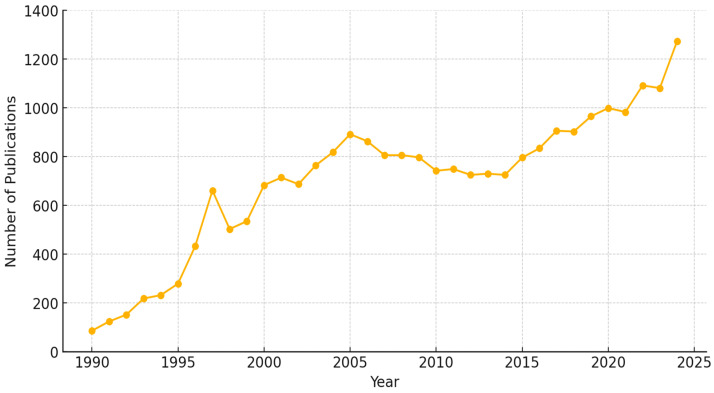
Total number of publications related to PV EL imaging over time, based on the Scopus reference index.

Based on the Scopus reference index, Figure 2 illustrates the total number of publications related to PV EL imaging over time. The data indicate a significant rise in research activity, particularly from the late 1990s onward, reflecting growing interest and advancements in this field. Notable fluctuations are observed, but an overall increasing trend suggests the expanding role of EL imaging in PV diagnostics and performance analysis.

## 2. Key Requirements for Electroluminescence Imaging

### 2.1. Physical and Operational Requirements for EL Imaging

EL imaging relies on precise operational and environmental conditions to ensure the accuracy and reliability of captured data. A fundamental requirement is the application of an electrical current to the PV module, which induces the emission of near-infrared light, detectable by a specialized imaging camera sensitive in the near-infrared (NIR) or shortwave infrared (SWIR) part of the light spectrum [6]. Key considerations for successful EL imaging include power supply stability, camera sensitivity, ambient conditions, and the structural integrity of the test object.

Firstly, the power supply must be capable of delivering sufficient forward bias current and voltage to the PV module to stimulate electroluminescence without causing damage. PV modules are typically forward biased to a certain voltage (*V_bias_*) that is equivalent to the short circuit current (*I_sc_*) of the module. This bias voltage needs to be larger than the open circuit voltage of the module (*V_oc_*). For example, a typical new-generation PV module with a rated power of 630 W and an open-circuit voltage of around 56 V would require a current source capable of supplying a forward bias of approximately 15 A and a voltage of 65 V to induce electroluminescence. This typically necessitates a well-regulated current source, as even slight variations in current or voltage can affect the uniformity of the emitted light and, consequently, the quality of the captured image.

Camera sensitivity is another critical factor. EL imaging cameras must be capable of detecting low-intensity infrared light, typically in the near-infrared spectrum between 900 nm and 1300 nm, corresponding to the bandgap emissions of crystalline silicon solar cells, whose peak is at approximately 1150 nm [40]. Modern systems often employ high-sensitivity charge-coupled device (CCD) or complementary metal-oxide semiconductor (CMOS) sensors, with quantum efficiencies exceeding 70% in this wavelength range. To ensure the clear detection of faint emissions, these cameras often achieve noise levels below 10 electrons per pixel and dynamic ranges exceeding 70 dB [41], enabling the resolution of subtle intensity variations that are critical for identifying microcracks and other defects.

**Figure 3:**
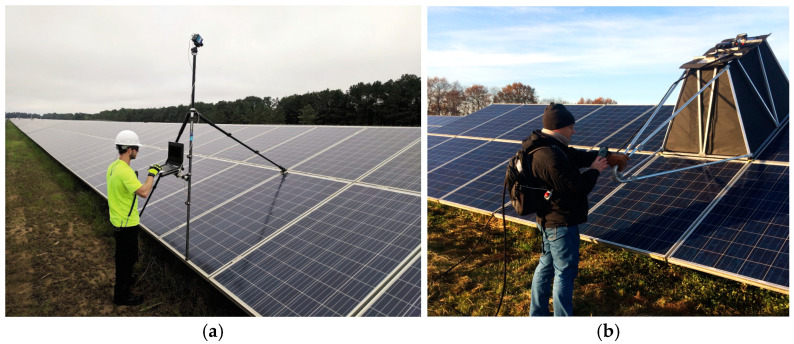
EL imaging setups: (**a**) EL imaging setup using a portable EL camera, typically employed during dusk, low irradiance periods, or nighttime conditions to minimize ambient light interference (https://www.pv-magazine-india.com/2020/05/02/the-long-read-el-to-the-field-assessing-plant-health/ (accessed on 24 March 2025)); (**b**) EL imaging inspection under sunlight, utilizing a protective box to create a dark environment and mitigate the effects of ambient illumination (https://isfh.de/fidelitas-en/?lang=en# (accessed on 24 March 2025)).

Environmental conditions play a pivotal role in the effectiveness of EL imaging, particularly in outdoor applications. Factors such as ambient light, moving clouds, and weather conditions can significantly influence the quality and interpretability of the captured images. Ambient light, especially sunlight, introduces substantial noise into EL imaging, as the emitted infrared light from the module is inherently faint [42,43]. For instance, Figure 3a shows an example of an EL imaging setup using a portable EL camera during low-light conditions, such as dusk or nighttime, to minimize ambient light interference. In such setups, the reduced natural light allows the faint infrared emissions to be captured more accurately. Additionally, outdoor setups often employ light-blocking materials, such as opaque shields or tents, as shown in Figure 3b, to create controlled environments. Advanced image-processing algorithms are frequently utilized to enhance the signal-to-noise ratio, allowing accurate defect detection even under challenging conditions. These algorithms leverage techniques such as adaptive filtering and Fourier transform-based noise reduction to isolate the electroluminescent signal from background interference, making them invaluable for field applications [44,45,46].

**Figure 4:**
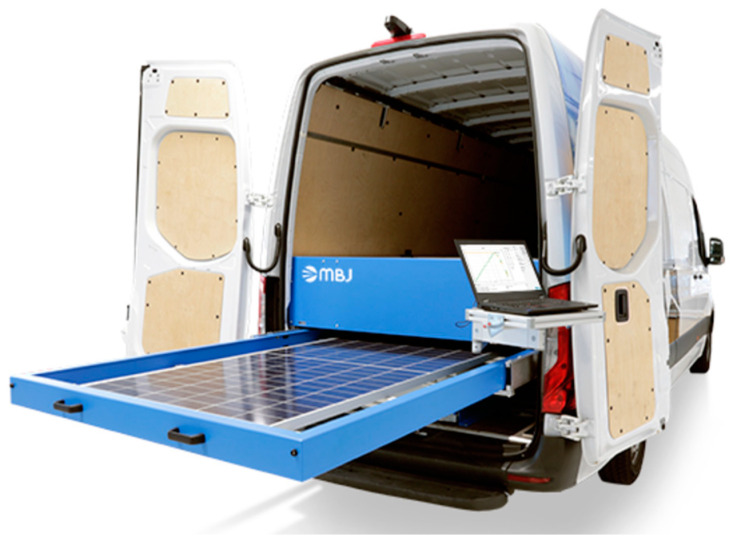
Mobile EL imaging solution by MBJ: A portable outdoor system for on-site PV module diagnostics. Source: https://www.mbj-solutions.com/en/products/equipment/mobile-lab (accessed on 24 March 2025).

Mobile EL solutions have recently emerged in the market as innovative tools for on-site diagnostics of PV modules, addressing the need for portable and efficient inspection systems. The mobile EL system developed by MBJ exemplifies this advancement, as shown in Figure 4, offering a fully integrated solution housed within a van. The setup includes a retractable platform for securely positioning PV modules, a protective enclosure to shield the imaging process from ambient light interference, and a high-resolution EL imaging device capable of detecting microcracks, degradation patterns, and other defects. The system also features a user-friendly interface with a laptop for real-time data acquisition and analysis, enabling quick and accurate diagnostics in outdoor environments. This solution is particularly advantageous for large-scale PV installations, reducing the downtime and logistical challenges associated with traditional indoor EL imaging.

**Figure 5:**
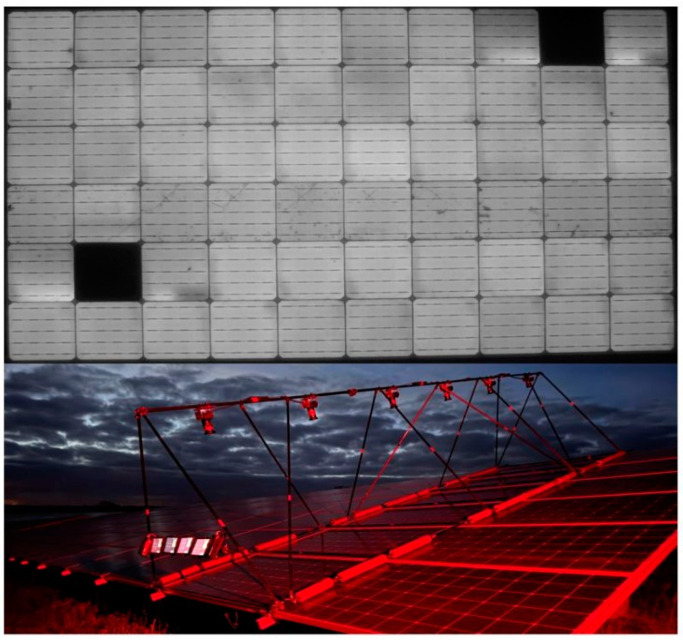
EL imaging solution by Aerial PV Inspection GmbH. The top image shows an example of a PV module with two blacked-out solar cells, indicating complete failure in those cells. The bottom image illustrates the outdoor nighttime EL imaging setup, which includes six cameras capable of simultaneously capturing multiple modules.

In contrast, indoor imaging benefits from highly controlled environments, where lighting and temperature can be maintained at optimal levels. However, outdoor EL imaging is indispensable for large-scale PV installations, where dismantling modules for indoor analysis is impractical. The development of robust shielding techniques (such as water/rain proofing), adaptive algorithms, and portable imaging equipment has been crucial in expanding the applicability of EL imaging to outdoor environments, even in less-than-ideal conditions. An excellent example of outdoor nighttime EL imaging is the solution developed by Aerial PV Inspection GmbH (Figure 5), which utilizes six cameras to simultaneously capture electroluminescence images from multiple modules. This setup significantly reduces inspection time while maintaining high spatial resolution and defect detection capabilities, demonstrating the feasibility and efficiency of large-scale outdoor EL inspections under low-light conditions.

### 2.2. Instrumentation and Imaging Equipment Essentials for Indoor EL

**Figure 6:**
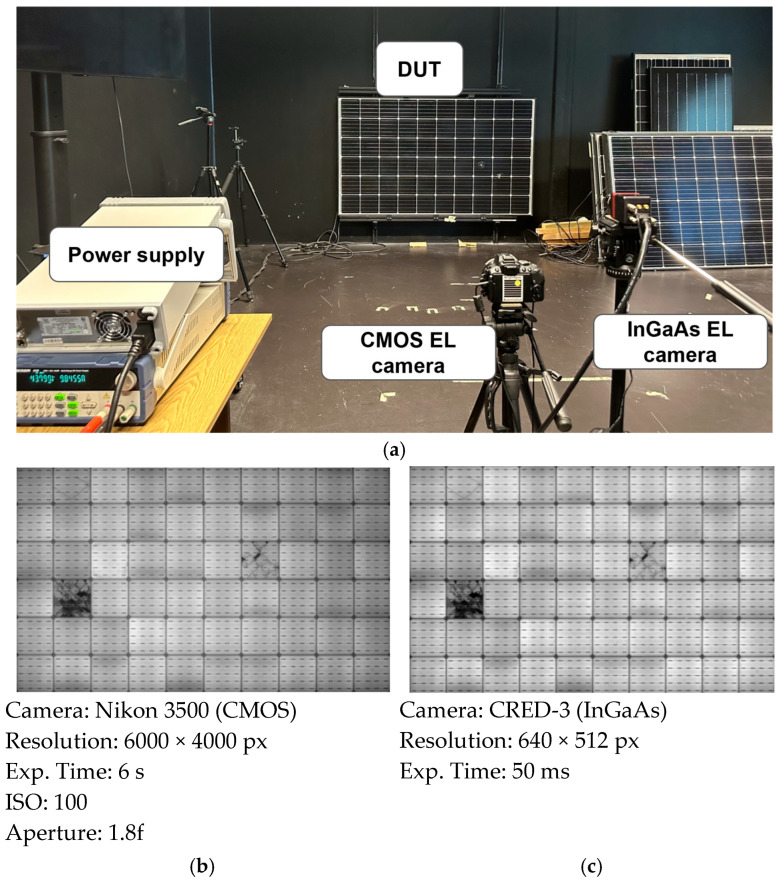
(**a**) Example of an EL imaging indoor setup with the main components labeled. (**b**) EL image of a PV module using a CMOS camera. (**c**) EL image of a PV module using an InGaAs camera.

Indoor EL imaging requires specific components to ensure the consistent acquisition of high-quality EL images. A typical setup is depicted in Figure 6, where an EL experiment was carried out. The key components of the setup are as follows:

- Module Stand: For indoor EL imaging, a stable setup that ensures a consistent module position is essential. Figure 6 illustrates an example of a wall-mounted frame used to secure the test modules, ensuring a perpendicular module-to-camera perspective. The rail is adjustable to accommodate different module sizes.
- Camera: The camera setup, as shown in Figure 6, consists of an EL-compatible camera mounted on a tripod. The camera should be positioned as perpendicularly as possible to the module, with the tripod allowing adjustments to different heights. The focus must be set to achieve sharp image quality for defect visualization. Two types of cameras are commonly used for EL imaging:
  ○ CMOS Cameras (with the IR filter removed): These cameras capture EL emissions up to 1100 nm and typically offer high resolution. They require longer exposure times (several seconds), depending on the module technology, as well as tunable ISO and aperture settings.
  ○ InGaAs Cameras: These cameras operate within the 950–1300 nm range, which perfectly overlaps with the emission spectrum of crystalline silicon. As a result, they require shorter exposure times for EL imaging. However, they generally have lower resolution compared to CMOS cameras.
- Power Supply Unit (PSU): A DC power supply is used to forward bias the PV module, generating the EL signal necessary for imaging.
- Camera Remote Control: Software control of the camera allows for the precise adjustment of ISO, exposure time, and aperture settings, ensuring high-quality EL image acquisition.

EL images often require post-processing to correct for certain problems that could arise in acquisition; firstly, proper camera focus is required, and then, vignetting and lens distortion from the camera needs to be corrected [47]. Furthermore, proper camera calibration is often required to remove offset pixel values; some cameras require dark bias calibration and flat-field correction [48]. A dark bias calibration consists of acquiring a completely dark image with the same exposure time as the intended EL image. By subtracting the dark background image from the EL image, one can eliminate stray light from the setup, camera noise, and dead camera pixels. Flat-field correction can be applied to correct for non-uniform lighting in the image or patterns such as vignetting; this consists of acquiring an image of a bright homogeneous surface close to the camera and dividing the EL image by it.

When the camera perspective is not perfectly perpendicular, and the camera field of view also covers part of the background, image perspective correction, module detection, edge and corner extraction, and cropping need to be performed [49,50,51,52,53,54,55]. More advanced methods based on machine learning can accelerate the processing process [56].

### 2.3. Challenges in Indoor and Outdoor EL Imaging

As summarized in Table 1, EL imaging faces distinct challenges depending on whether it is conducted indoors or outdoors, as well as on shared obstacles that apply across both environments. Indoor EL imaging, while benefiting from controlled conditions, is hindered by practical issues such as the difficulty of handling large PV modules. Additionally, the cost of setting up dedicated high-resolution imaging facilities can be prohibitive for smaller laboratories.

Outdoor EL imaging must contend with dynamic and often unpredictable environmental conditions. In nighttime conditions, ambient light interference, particularly from moonlight or artificial sources, presents a challenge, requiring image-processing algorithms to enhance the signal-to-noise ratio. Adverse weather conditions, such as rain or wind, can disrupt the imaging process and destabilize equipment. Furthermore, dark inspection poses safety concerns due to the people involved having to work with electrical equipment during the night. Moreover, the logistical effort required to inspect large-scale PV installations efficiently necessitates portable and robust imaging solutions.

Outdoor daylight EL imaging presents even greater challenges due to high ambient light levels, which significantly reduce contrast and necessitate advanced filtering techniques to isolate EL signals. Shorter exposure times are required to prevent overexposure, limiting signal detection. Additionally, current daylight-compatible EL imaging systems are less mature and often expensive, making large-scale deployment difficult. Power supply constrains further complicate outdoor imaging, as stable and portable power sources are essential for reliable operation. Finally, extensive post-processing is required to extract meaningful EL data, adding to the complexity of daylight EL imaging workflows.

Shared challenges, such as achieving sufficient resolution for detecting microcracks, managing the large volumes of data generated, and ensuring electrical safety during high-current injections, further complicate the process. Addressing these issues is critical for improving the reliability and scalability of EL imaging, and ongoing advancements in technology, including AI-driven solutions and enhanced imaging hardware, are expected to play a significant role in overcoming these barriers.

## 3. Recent Advancements in Electroluminescence Imaging: Innovations of Outdoor Daylight EL Imaging

### 3.1. Hardware Innovations for Daylight EL Imaging

**Figure 7:**
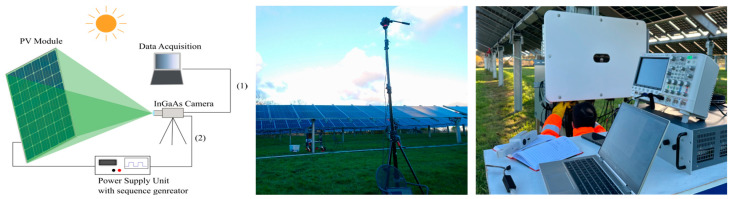
Schematic representation of a daylight EL imaging setup for PV modules.

Daylight EL imaging has emerged as a powerful tool for real-time diagnostics of PV modules under outdoor illumination conditions. Unlike indoor EL imaging techniques that require complete darkness, daylight EL imaging enables defect detection without the need for specialized shading setups, significantly enhancing its applicability in large-scale field inspections [4,57,58,59,60]. Figure 7 illustrates the hardware setup for daylight EL imaging.

The PV module is illuminated by sunlight while an external power supply unit (PSU) injects the required current into the PV module. An indium gallium arsenide (InGaAs) camera captures the EL emissions, with data acquisition being performed through a connected computer. Two distinct connections are shown:

- Connection step (1): The InGaAs camera directly captures EL emissions under ambient sunlight, highlighting the versatility of the setup in adapting to varying outdoor lighting conditions.
- Connection step (2): A secondary connection from the PSU to the PV module ensures stable current injection when producing the EL signal. Present-day PSU’s have built-in sequencers or function generators that can be used to program a modulated current signal to the PV string. Alternatively, oscilloscopes or switching boxes can be used to create the modulated current sequence.

**Figure 8:**
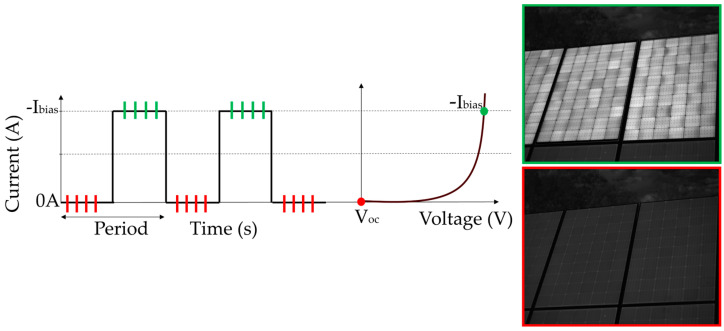
Methodology and example output of daylight EL imaging for PV modules. The process involves biasing the module with a periodic waveform (e.g., 30 Hz) to alternate between EL-active (current ON) and background-only (current OFF) states. EL and background images are captured and averaged to enhance the signal-to-noise ratio (SNR). The resulting processed image isolates EL emissions, enabling accurate defect detection under outdoor conditions.

Figure 8 presents a schematic representation of a widely adopted approach for daylight EL imaging, showcasing the key steps and example outputs. This method has gained significant attention due to its ability to enhance the signal-to-noise ratio (SNR) and enable EL imaging under outdoor illumination. The methodology involves the following steps:

- Periodic Current Injection for Modulation: Daylight EL imaging techniques commonly utilize a periodic current waveform, typically in the range of 20–50 Hz, with 30 Hz being a frequently cited value in the literature. This waveform ensures that the EL signal can be effectively distinguished from the ambient light background. The alternating ON and OFF states of the current allow clear separation between EL-active images and background-only images [3,4,61].
- Simultaneous Acquisition of EL and Background Images: Advanced imaging systems, often equipped with InGaAs cameras, capture both EL and background images during the modulation process. During the ON phase of the current, the camera captures the EL emission from the PV module, while during the OFF phase, it records only the ambient background. This dual-image acquisition facilitates robust background subtraction in subsequent processing stages.
- Signal Averaging to Enhance SNR: To mitigate noise and improve the clarity of EL images, several studies recommend averaging multiple frames of both the ON and OFF states of sequential frames in the same modulation period. This averaging process reduces random noise and significantly enhances the SNR, a critical factor for accurate defect detection under high ambient light conditions.
- Processed Output for Defect Identification: The processed output, obtained by subtracting the averaged background image from the averaged EL image, results in a clean and high contrast EL image. As shown in the example of Figure 8, this method effectively highlights module defects, such as microcracks, inactive areas, and degradation patterns, even in challenging daylight environments.

### 3.2. Why InGaAs Cameras Are Most Suitable for Outdoor EL Imaging

**Figure 9:**
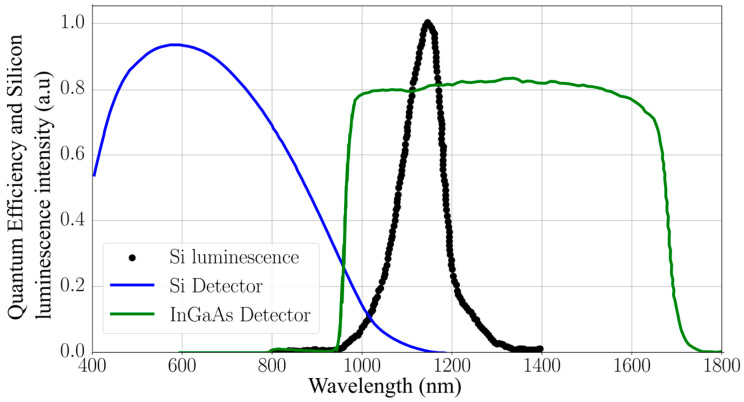
Quantum efficiency of InGaAs detectors compared with silicon-based detectors, highlighting their overlap with the electroluminescence emission spectrum of crystalline silicon PV modules. The high QE of InGaAs detectors in the NIR range combined with their ability to operate under short exposure times makes them ideal for outdoor EL imaging by suppressing sunlight interference and enhancing defect detection.

The key advantage of InGaAs cameras lies in their high quantum efficiency (QE) in the near-infrared (NIR) range, as presented in Figure 9, aligning closely with the EL emission spectrum of crystalline silicon (c-Si) PV modules, which peaks at approximately 1150 nm. This spectral overlap allows InGaAs cameras to efficiently detect EL emissions while ignoring much of the visible light spectrum, where sunlight interference is strongest. This inherent sensitivity in the NIR region makes InGaAs cameras far superior to silicon-based detectors, which have limited sensitivity beyond 900 nm and are more affected by visible light.

Another critical feature of InGaAs cameras is their ability to operate with extremely short exposure times in the millisecond range. Short exposure times are vital for outdoor EL imaging because they reduce the risk of sensor saturation caused by intense sunlight. By rapidly capturing multiple frames and averaging the results, InGaAs cameras achieve a high SNR, which is crucial for accurately detecting defects in PV modules under daylight conditions. This capability is particularly advantageous when combined with visible light filters that block unnecessary wavelengths, further minimizing interference from sunlight.

InGaAs cameras are also highly versatile in handling varying outdoor lighting conditions, from full sunlight to overcast skies. Their robust NIR sensitivity ensures consistent performance, enabling precise defect detection across a range of environmental scenarios.

### 3.3. The Use of Optical Filters in Daylight Luminescence Imaging

The main challenge of daylight EL inspections is the significantly lower signal that the PV modules have relative to the ambient sunlight. To address this problem, optical filters are used to filter out the sunlight surrounding the silicon emission. These filters help improve the SNR of the resulting images during the inspections.

There are multiple options for filtering, ranging from long-pass to bandpass filters, usually centered around the silicon emission peak (1150 nm) and with different bandwidths. Table 2 shows examples of different filter setups used in different daylight luminescence inspections.

**Figure 10:**
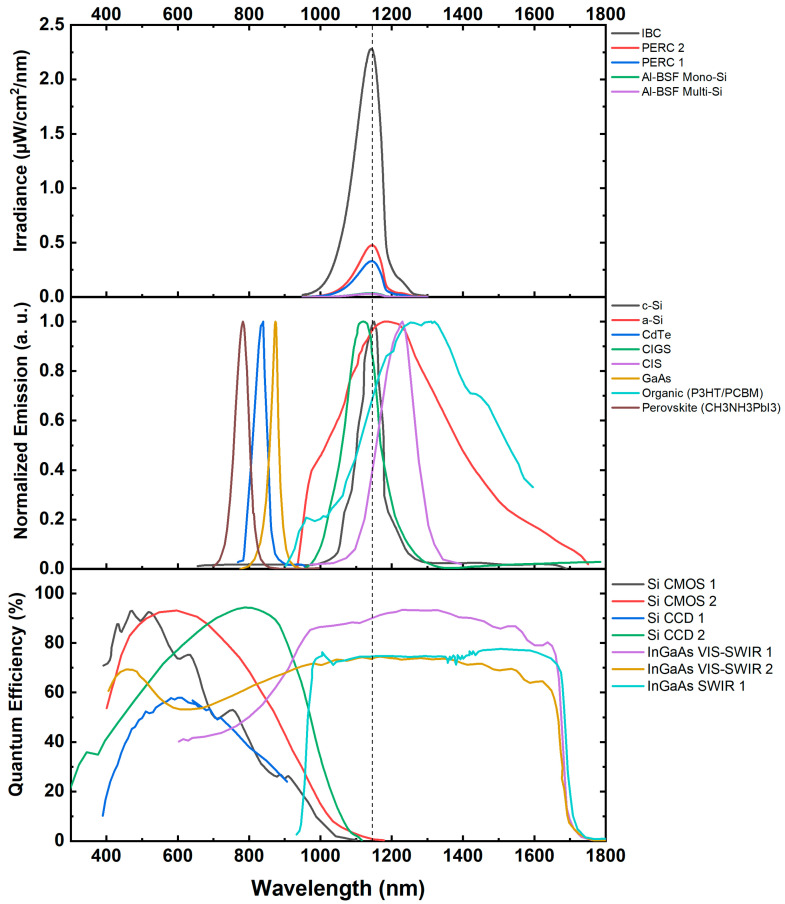
(**Top**): Spectra of five PV modules of different technologies in linear scale [40]. (**Middle**): Normalized reflected emission spectra of a selection of PV technologies. (**Bottom**): Quantum efficiency spectra of a selection of commercially available camera sensors.

On top of the optical filters, neutral density filters and a lens with an iris were tested in daylight inspections in order to reduce the intensity of the sunlight signal and improve the SNR [70]. When exploring daylight EL imaging, understanding the module technology under inspection is crucial. Because of the exponential relationship between EL intensity and cell diode voltage, newer cell technologies with larger voltages will have more intense luminescence peaks (as shown in Figure 10); for example, cell technologies such as IBC can reach emission intensities of over four times larger than those of PERC and Al-BSF. This is especially crucial in daylight inspections due to the effect of sunlight diminishing the signal captured by the camera. Previous research [40] investigated the reflected spectra of cells in PV modules of Al-BSF, PERC, and IBC. Modules with IBC cells, which had higher voltages, showed much larger luminescence peaks, making them suitable for daylight EL inspection without electrical modulation. These results are especially important for estimating the duration of daylight inspections; when inspecting plants with new-generation modules, the acquisition time can be reduced because of the larger signal intensity.

Large utility-scale PV plants primarily consist of crystalline silicon modules; however, other PV technologies, such as cadmium telluride (CdTe) and perovskites, exhibit different emission peaks compared to silicon. Throughout this review, emphasis has been placed on CMOS and InGaAs cameras due to their suitability for imaging silicon-based modules. However, when imaging other PV technologies, careful consideration must be given to the selection of camera sensors and optical filters to ensure compatibility with the emission characteristics of the specific technology. Figure 10 illustrates the emission peaks of various PV technologies alongside the quantum efficiency spectra of selected commercial camera sensors.

### 3.4. Field Application of Daylight EL: Case Study

**Figure 11:**
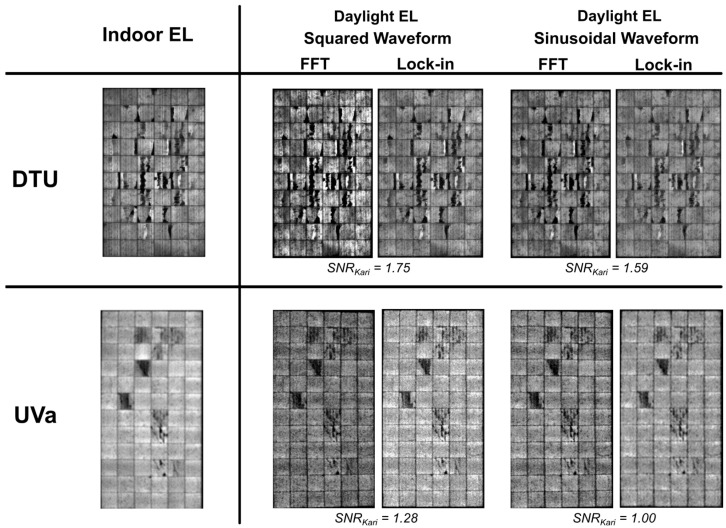
Comparison of daylight EL imaging results from case studies conducted at DTU (552–560 W/m^2^) and UVa (750 W/m^2^) with an InGaAs camera with 10 ms exposure time and 32 fps framerate. Different waveforms (square and sinusoidal with 4 Hz modulation frequency) and signal processing methods (Fast Fourier Transform and lock-in) are evaluated. The SNR values indicate the superior performance of square waveforms and lock-in processing, particularly under lower irradiance conditions at DTU. Results from UVa demonstrate a decline in the SNR due to higher ambient light interference [59].

Several studies have explored the practical implementation of daylight EL imaging to evaluate PV module performance under varying outdoor conditions. The effectiveness of daylight EL imaging depends on factors such as irradiance levels, waveform types for current modulation, and signal processing methods, all of which influence the SNR and the quality of the resulting EL images. Figure 11 illustrates comparative results from case studies conducted at two different facilities: the Technical University of Denmark (DTU) and the Universidad de Valladolid (UVa) [59].

At DTU, daylight EL imaging was performed under outdoor conditions with an irradiance range of 552–560 W/m^2^. Two modulation waveforms were tested: square and sinusoidal. The square waveform exhibited a slightly higher SNR (1.75) compared to the sinusoidal waveform (1.59) when processed with lock-in techniques, suggesting that the square wave modulation higher average current injection is more advantageous for obtaining clear images. Fast Fourier Transform (FFT)-based processing provides images with better contrast in general. The results demonstrate the critical role of advanced signal processing methods in isolating the EL signal from ambient noise during daylight imaging.

At UVa, a similar methodology was employed under higher irradiance conditions (750 W/m^2^). The comparative analysis showed a decline in the SNR (maximum 1.28 with square waveform and lock-in processing) compared to the results from DTU, which can be attributed to the increased ambient light interference at higher irradiance levels. The sinusoidal waveform exhibited further reductions in the SNR (1.00), highlighting its lower efficacy under intense sunlight. This emphasizes the importance of selecting appropriate waveform types and image processing techniques tailored to specific outdoor conditions to maximize the effectiveness of daylight EL imaging. These processing techniques are based on the separation of the EL signal from the background in the modulation sequence; different research investigates the application of these computer methods [44,71].

**Figure 12:**
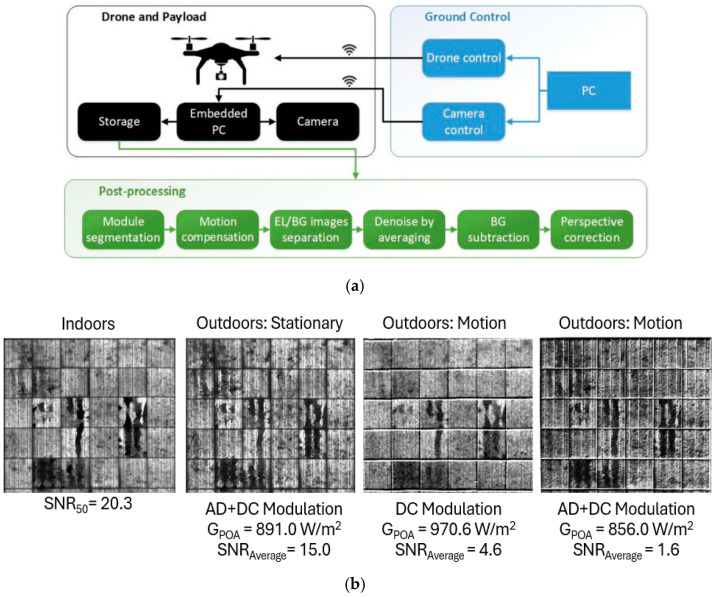
(**a**) Drone-based daylight EL imaging system architecture; (**b**) example EL images captured in various conditions using a drone-based system. Images include indoor scenarios, stationary outdoor setups, and drone-based outdoor imaging during motion [3].

Additionally, drone-based daylight EL imaging represents a cutting-edge innovation in the field of PV diagnostics, providing a scalable and efficient solution for inspecting large PV plants. Unlike stationary imaging setups, drones equipped with advanced EL imaging systems can cover vast areas with reduced effort from the operators, significantly reducing inspection time and costs [72,73,74]. Figure 12a,b illustrate the architecture of a drone-based EL imaging system and example EL images captured under various conditions.

As shown in Figure 12a, the drone system integrates several critical components: a camera for capturing EL images, an embedded PC for processing and storing data, and wireless communication modules for real-time control and data transmission to the ground station. The drone communicates with the ground control unit, which handles both drone navigation and camera operations. This setup ensures the precise alignment of the camera with the PV module, even during motion. After image acquisition, post-processing steps such as module segmentation, motion compensation, EL and background (BG) image separation, denoising by averaging, and perspective correction are performed to produce high-quality EL images. These processes are vital for mitigating challenges such as drone movement and varying sunlight conditions during outdoor inspections.

Figure 12b showcases EL images captured under various scenarios—indoors, outdoors (stationery and motion), and with different modulation techniques. The corresponding SNR and global plane of array irradiance (G_POA_) values provide quantitative insights into the performance of this method under diverse conditions. Indoor EL imaging achieves the highest SNR (SNR_50_ = 20.3) due to the controlled lighting environment, which eliminates external sunlight interference. This allows for the capture of detailed EL images with minimal noise, providing an ideal baseline for comparing outdoor performance. In contrast, outdoor imaging introduces challenges, as ambient sunlight significantly impacts the ability to distinguish the EL signal from the background.

For outdoor stationary imaging, the use of AC+DC modulation, with G_POA_ = 891.0 W/m^2^, yields an average SNR of 15.0. While lower than the indoor scenario, this result demonstrates that advanced modulation techniques effectively suppress noise and retain sufficient detail for defect detection. AC+DC modulation, which combines alternating and direct current injections, ensures that the EL signal remains discernible even under strong sunlight conditions.

Drone-based EL imaging during motion presents additional complexities. When using only DC modulation (G_POA_ = 970.6 W/m^2^), the SNR drops significantly to 4.6, as motion-induced artifacts and sunlight interference degrade image quality. However, employing AC+DC modulation improves the robustness of the method, with G_POA_ = 856.0 W/m^2^, though the SNR decreases further to 1.6. These results underscore the difficulty of maintaining a high SNR during motion and highlight the need for further advancements in motion compensation and noise reduction techniques.

**Figure 13:**
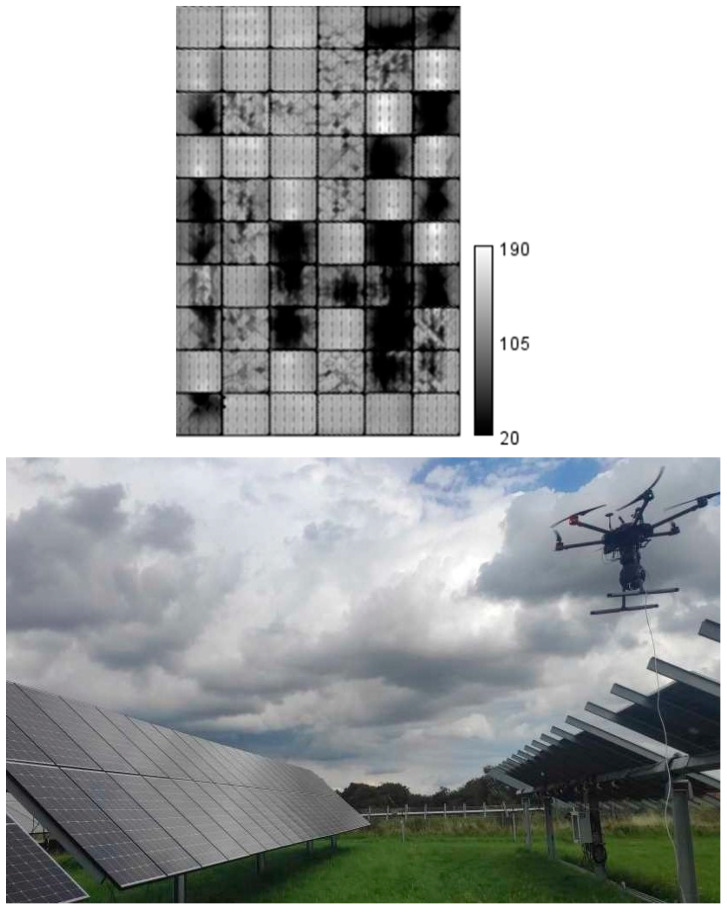
Example of an EL image acquired in a daylight EL inspection conducted using a drone at the DTU outdoor PV testing facility [68].

Another research has investigated in more detail the challenges and difficulties of daylight drone-based EL imaging compared to stationary EL inspections [68]. Motion from the camera carrier has a big impact on the difficulty of extracting the EL signal from the images, where PV module recognition is required in all frames of the EL recording for proper alignment. Because of that, a proper camera calibration to remove offsets and image aberrations is essential. Furthermore, when employing drones for EL imaging, care must be taken to avoid casting a shadow over the modules, since it would affect the image post processing. Figure 13 shows an example of a daylight drone-based EL inspection and a resulting image of a defective module in the string. In very defective modules, large cracks could be easily detected; however, some contrast is lost because of the sunlight, and information is lost for small defects such as microcracks due to motion blur.

## 4. AI-Driven Perspectives in EL Imaging

### 4.1. Introduction to AI in EL Imaging

The years 2022 to 2024 mark a significant period in the evolution of AI-driven methodologies for automating defect detection and segmentation in PV systems using EL imaging. According to Scopus search results, nearly 135 research papers specifically addressing AI-based automation for EL defect detection were published during this timeframe. This surge in publications underscores the growing interest in applying artificial intelligence to PV quality control, driven by advancements in deep learning architectures, novel datasets, and the increasing deployment of PV systems worldwide.

**Figure 14:**
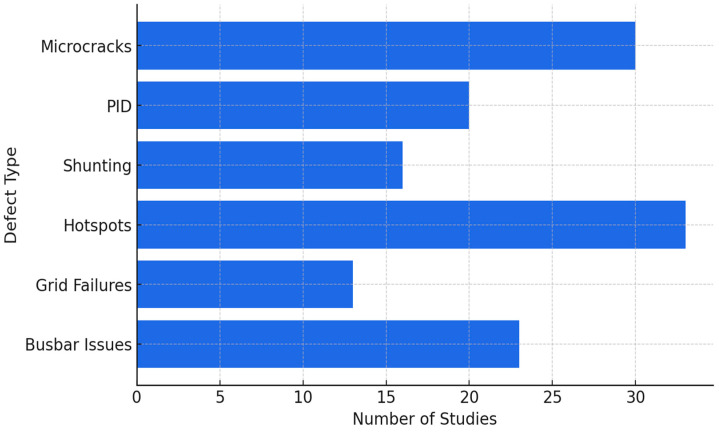
Distribution of defect types studied in AI-driven EL imaging research from 2022 to 2024.

A detailed review of these publications reveals that microcracks, hotspots, PID, busbar issues, and shunting are the most frequently studied defects, as illustrated in Figure 14. The majority of studies focus on mono-Si and poly-Si PV modules, with fewer investigations addressing heterojunction and thin-film technologies. Additionally, a distinction is observed between studies analyzing individual cells versus full modules, highlighting the range of scales at which AI-based EL analysis is applied. However, a notable observation is the overlap within these 135 publications. Many were authored by the same research groups, and several journal papers were extensions or refinements of earlier conference proceedings. This highlights the need to distill the most impactful contributions, ensuring a balanced and focused review of the state-of-the-art methodologies during this period.

To provide a comprehensive and meaningful analysis, we reviewed 30 peer-reviewed papers [75,76,77,78,79,80,81,82,83,84,85,86,87,88,89,90,91,92,93,94,95,96,97,98,99,100,101,102,103,104,105,106,107,108], selecting 10 papers per year to ensure balanced representation. The selection was guided by three primary criteria: (1) their novel algorithms and methodologies; (2) the accuracy and robustness of the models; and (3) the datasets used in the studies. A significant portion of the reviewed studies utilized widely recognized datasets, such as the ELPV dataset, the PVEL-AD dataset, benchmark EL images, and UCF EL Defect, as summarized in Table 3. The evaluation focused on model performance metrics, including F1-score, and computational efficiency, as well as the scalability and practicality of the proposed solutions. For a detailed explanation of the metrics and terminologies used in this section, see Appendix A.

### 4.2. Advancements in AI for Solar PV Defect Detection and Segmentation (2022)

**Figure 15:**
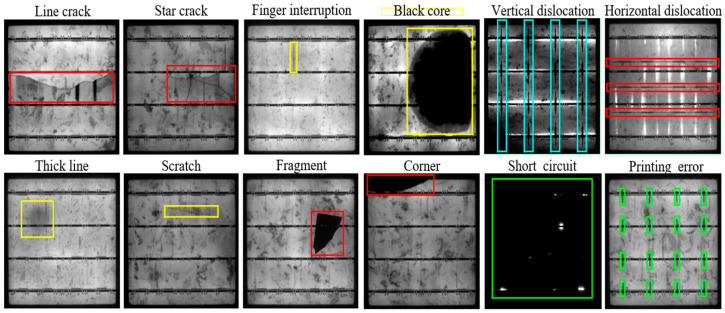
Examples of defect types from the PVEL-AD dataset [75]. The dataset includes various defect categories commonly observed in PV modules, such as line cracks, star cracks, finger interruptions, black cores, vertical and horizontal dislocations, thick lines, scratches, fragments, corners, short circuits, and printing errors. Each defect type is highlighted with bounding boxes in different colors for clarity, illustrating the diversity and complexity of anomalies present in PV modules.

The year 2022 marked substantial advancements in AI methodologies for defect detection and segmentation in PV cells, showcasing innovations in datasets, architectures, and algorithms tailored to address emerging challenges. Among the most impactful contributions was the introduction of the PVEL-AD dataset (see Figure 15), a benchmark dataset comprising an array of labeled EL images encompassing various defect types [75]. This dataset became instrumental in validating AI-driven methodologies, including models like SeMaCNN [76], which employed Mahalanobis distance-based segmentation. By leveraging this statistical approach, SeMaCNN achieved a remarkable accuracy of 94.6% and an F1-score of 91.1%, demonstrating its capability to handle noisy industrial datasets effectively.

Another transformative area of progress was the adoption of transfer learning techniques, which addressed critical challenges such as class imbalance and domain adaptation. For instance, the Logit Inducing with Abnormality Capturing (LIAC) [77] method incorporated transfer learning with a novel logit-inducing loss function to improve segmentation performance across datasets with heterogeneous defect distributions. Models like ERDCF-Net [78], with their lightweight architecture optimized for real-time segmentation, complemented these innovations by focusing on efficiency without compromising accuracy. ERDCF-Net’s design highlighted an industry-aligned emphasis on computational feasibility for large-scale deployment. These approaches collectively underscored the importance of balancing high accuracy with scalable implementations—a recurring theme throughout 2022.

The role of attention mechanisms emerged as a critical enabler of precision in defect detection. For example, the Attention Classification-and-Segmentation Network integrated transfer learning with attention modules, enhancing feature extraction for micro-crack detection. This method demonstrated remarkable precision by selectively emphasizing defect regions in high-dimensional feature spaces, a feature that general architectures often struggle to recognize [79]. Similarly, Gradient-Guided Architectures adopted filter-tuning techniques to overcome data scarcity, enhancing both feature generalization and segmentation accuracy [80]. These methods paved the way for adaptive feature extraction, improving model performance in scenarios with limited or imbalanced datasets.

**Figure 16:**
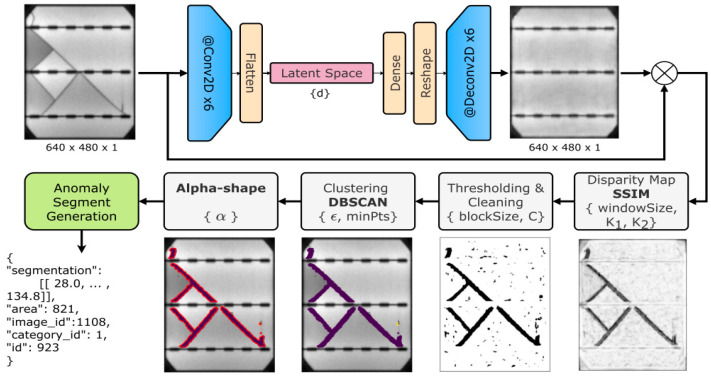
Anomaly detection and segmentation pipeline leveraging latent space encoding and advanced clustering methods [82].

The evolution of You Only Look Once (YOLO)-based architectures further contributed to this year’s advancements. Innovations like YOLO-PV tailored YOLO’s object detection capabilities to the domain of PV defect identification. By incorporating scalable annotation frameworks, YOLO-PV not only achieved a high average precision (AP) of 94.55%, but also delivered real-time applicability at 35 frames per second [81], significantly reducing manual labeling costs by 60%. In parallel, Figure 16 illustrates an advanced anomaly detection and segmentation pipeline developed by [82], which employs convolutional layers, latent space encoding, and clustering techniques like DBSCAN to enhance defect segmentation. This approach demonstrates the integration of mathematical and computational methods for precise defect detection in PV solar cells.

Comparative studies also played a pivotal role in 2022. For instance, models like ResNet, Xception, and Vision Transformer were benchmarked on augmented datasets, revealing the critical importance of architecture selection in achieving optimal performance. Among these, Xception demonstrated superior accuracy (91.4% on ELPV dataset) [83], highlighting its ability to align well with complex dataset characteristics. The integration of real-world and augmented datasets, including aerial EL imaging for large-scale PV plant monitoring, further emphasized the importance of practical deployment scenarios [84].

Despite these breakthroughs, several challenges remain. Benchmark datasets like PVEL-AD, while valuable, often fail to capture the full spectrum of real-world conditions, such as module-level degradation influenced by environmental factors like temperature, humidity, and irradiance variability. Lightweight architectures, such as ERDCF-Net, have shown promise for real-time applications, yet their robustness in industrial-scale deployment under diverse operating conditions remains underexplored. Moreover, most efforts have concentrated on cell-level defect detection, leaving critical gaps in addressing module-level integration—a necessity for commercial scalability.

Additionally, the trade-off between computational efficiency and model complexity persists, particularly for edge deployments in resource-constrained environments. While transfer learning and attention mechanisms have proven effective for micro-crack and anomaly detection, there is a need for greater generalization across defect types and datasets. Future directions should prioritize adaptive models capable of evolving with defect variability, integrating multi-modal imaging techniques such as thermal and EL imaging and developing diverse, open-access datasets that better reflect real-world operating challenges.

### 4.3. Advancements in AI for Solar PV Defect Detection and Segmentation (2023)

The year 2023 witnessed substantial progress in AI-driven methodologies for solar cell defect detection, reflecting a strong focus on improving model robustness, segmentation accuracy, and industrial applicability. Among the standout contributions was the development of SEiPV-Net, a sophisticated encoder–decoder network tailored for the segmentation of 24 distinct defect types in EL images. This network’s integration of custom class weights and weighted loss functions significantly enhanced its ability to detect smaller defect types often overlooked by conventional architectures. SEiPV-Net demonstrated superior performance, achieving high Intersection over Union (IoU) and F1-score values, outperforming established models like DeepLabv3+ and U-Net [86]. This work emphasized the importance of balancing precision with robustness when dealing with diverse defect morphologies.

A parallel focus on segmentation was evident in the application of dual spin max pooling convolutional neural networks (CNNs), which were optimized for detecting cracks, shading, and PID in solar cells. Achieving an impressive accuracy of 99.5%, this system validated its effectiveness through real-world thermal testing, reinforcing its industrial relevance. By focusing on defect types common in operational PV systems, the approach bridged the gap between research innovation and practical deployment [85]. These advancements highlighted the growing shift toward tailored architecture designed to address specific defect scenarios in PV cells.

**Figure 17:**
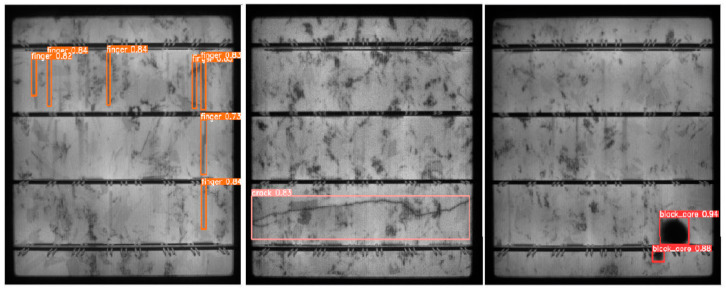
Example outputs from an optimized YOLOv5 model applied to defect detection in PV cells [88].

Transfer learning continued to be a transformative tool for overcoming domain adaptation challenges. A novel approach embedded adversarial learning and attention mechanisms to enhance cross-domain generalization, particularly for monocrystalline and polycrystalline solar cells. This technique achieved a precision of 90.15% and a recall of 84.7% when applied to polycrystalline datasets, showcasing its ability to adapt to varying imaging conditions and defect distributions [87]. Similarly, an optimized YOLOv5 model, utilizing advanced data augmentation techniques and attention mechanisms and achieving an mAP@0.5 of 96.1% on the ELPV dataset, was developed by [88], seeing an improvement of 10.38% compared with the original YOLOv5 model. As illustrated in Figure 17, the model effectively identifies defects such as fingers, cracks, and black cores, with bounding boxes and confidence scores emphasizing its precision in defect localization and classification. This improvement highlights the synergy between augmentation strategies and feature extraction in enhancing the model’s robustness and applicability in real-world scenarios.

The importance of benchmarked datasets was underscored by studies leveraging pixel-level annotated data for rigorous evaluations. For instance, one study introduced a dataset of 593 EL images with pixel-level ground truth masks for 24 defect types, enabling comparisons of semantic segmentation models like DeepLabv3+ and PSPNet [89]. The incorporation of custom class weights improved the detection of smaller and less frequent defect types, emphasizing the critical role of balanced datasets in training robust models. These datasets provided a foundation for testing and validating cutting-edge methodologies.

Another approach addressed micro-crack detection and classification using attention-enhanced CNNs [90], integrating real-world operational data with a novel feature weighting strategy. This approach achieved an F1-score of 87.5% and was particularly suitable for deployment in automated manufacturing lines. Meanwhile, advanced anomaly detection techniques, particularly those leveraging Mahalanobis distance, showed promise for detecting subtle defects in noisy datasets [91]. These techniques demonstrated F1-scores exceeding 91.1% using the ELPV dataset, validating their robustness.

The evolution of YOLO-based architectures remained prominent, with an enhanced YOLOv5 model incorporating Global Attention Modules and adaptive feature fusion [92], achieving a 2.5% mAP@0.5 improvement over the standard YOLOv5 framework. The addition of Test Time Augmentation further boosted its performance, making it a standout approach for defect detection in complex EL images. Lightweight architectures, such as custom CNNs optimized for rapid inference, achieved significant performance improvements while maintaining computational efficiency, showcasing their potential for real-time applications in resource-constrained environments [93]. An F1-score of 85% was achieved using this new methodology.

In addition, the integration of generative adversarial network (GAN)-based frameworks provided solutions for addressing data limitations. One study employed a hybrid GAN framework to generate synthetic training data, reducing class imbalance and improving model performance on rare defect types. This approach achieved an mAP@0.5 accuracy of 88.41% to 98.05% on augmented datasets, highlighting the importance of leveraging synthetic EL data [94]. The use of GANs underscores the growing recognition of the need for robust data augmentation techniques to complement advanced neural architectures.

Despite these advancements, several unresolved challenges persisted. The reliance on curated datasets, while beneficial for benchmarking, often limits model generalizability in real-world settings characterized by greater variability in defect types, imaging conditions, and environmental influences. While transfer learning and adversarial learning techniques showed promise in addressing domain shifts, their robustness across diverse imaging scenarios and defect morphologies remains underexplored. Similarly, lightweight architecture designed for real-time applications often faced trade-offs between computational efficiency and model accuracy, which must be addressed for large-scale deployment.

Moreover, although models like SEiPV-Net and enhanced YOLOv5 excelled in specific tasks, their integration into multi-stage systems for holistic PV module assessment is still in its infancy. Multi-stage frameworks capable of combining detection, classification, and root cause analysis are essential for achieving comprehensive quality control in industrial PV applications. Additionally, the exclusive reliance on EL imaging for most studies limits their ability to detect non-electrical defects, underscoring the need for multi-modal approaches that integrate thermal, visual, and EL imaging.

### 4.4. Advancements in AI for Solar PV Crack Detection and Segmentation (2024)

The year 2024 brought significant breakthroughs in AI-driven methodologies for PV cell defect detection and segmentation, characterized by innovative architectures, practical implementations, and the integration of advanced machine learning techniques. A standout contribution was the development of a CNN-based Transformer module featuring polarized self-attention, which excelled at distinguishing between spatial and semantic features. This model achieved a notable mAP@0.5 of 77.9% on the PVEL-AD dataset, highlighting its robustness in handling complex feature separations [95]. The introduction of PD-DETR, an object detection model tailored for PV defect segmentation, complemented this advancement. By leveraging dilated convolutions and adaptive hybrid matching, PD-DETR addressed challenges associated with intricate backgrounds and achieved an AP of 64.7% using the PVEL-AD dataset, demonstrating its capability to generalize across diverse imaging conditions [96].

**Figure 18:**
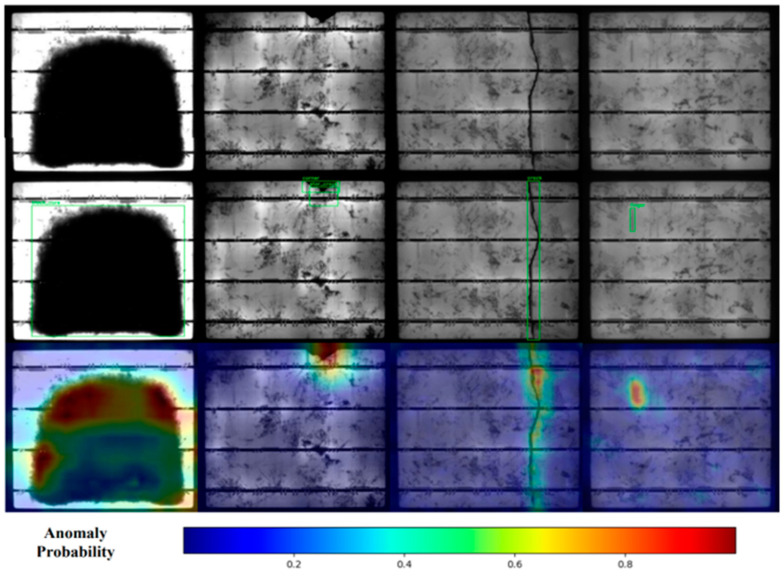
Visualization of anomaly detection using the Wave Flow model. The first row shows the EL image input, the second row represents the ground truth defect annotations, and the third row displays the predicted anomaly maps. The color scale, ranging from blue (low probability) to red (high probability), highlights defect regions such as micro-cracks and dislocations [99].

Wavelet analysis emerged as a novel approach to anomaly detection, marking a departure from conventional feature extraction techniques. The Wave Flow model, a wavelet-based normalized flow framework developed by [97], demonstrated exceptional accuracy in handling non-stationary textures in EL images, as illustrated in Figure 18. The figure depicts input EL images, ground truth annotations, and predicted anomaly maps, where the color-coded probabilities effectively highlight defects such as micro-cracks and dislocations, with Area Under the Receiver Operating Characteristic Curve (AUROC) scores of 94.4% (using the PVEL-AL dataset) and 98.0% (using the CSD dataset).

Lightweight architecture also gained traction in 2024, driven by the need for models that are deployable in resource-constrained environments. RAFBSD [98], a CNN-based framework optimized for edge devices, achieved a 5.3% improvement in mAP@0.5–0.95 compared to YOLOv8, balancing performance with computational efficiency. Similarly, a new model named LD-DETR further advanced this trend by focusing on reducing inference time without compromising detection accuracy [99], making it a viable candidate for real-time applications in industrial PV quality control. This technique achieved an AP of 87.4% and an inference time of 6.1 ms on GPU, as well as 1.1 s on the embedded CPU.

**Figure 19:**
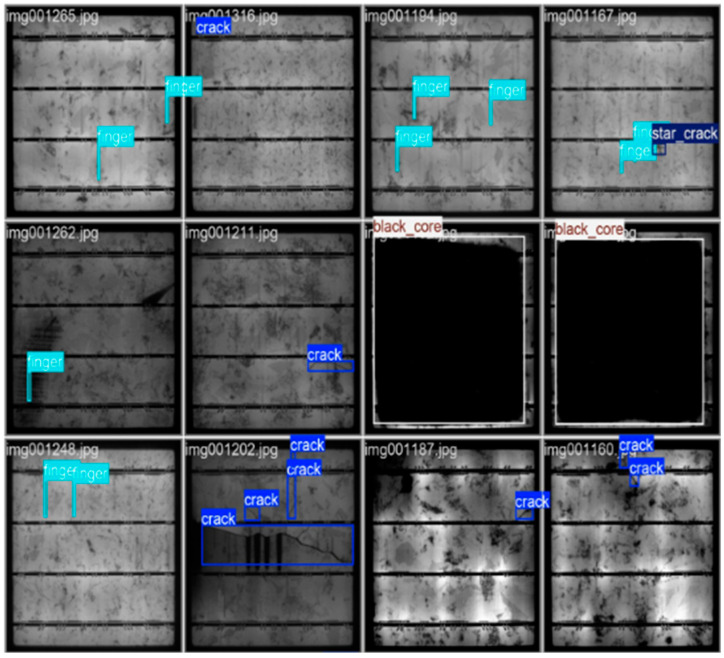
Output examples of defect detection using the YOLOv10 model [104].

The evolution of YOLO-based architecture continued to dominate the field. YOLOv10, enhanced with Compact Inverted Blocks and Partial Self-Attention [104], achieved an impressive mAP@0.5 of 98.5% on the PVEL-AD dataset, reinforcing its status as a benchmark for real-time defect detection. As illustrated in Figure 19, the YOLOv10 model effectively detects and localizes various defects, including cracks, fingers, star cracks, and black cores, further validating its robustness and precision in addressing complex defect detection tasks. Building on this, YOLO-ACF introduced adaptive complementary fusion mechanisms, resulting in a 2.3% improvement in mAP@0.5–0.95 over YOLOv8, further solidifying its capability to detect fine-grained defects in PV cells [100]. The adaptability of YOLO models continues to set the standard for defect detection frameworks, particularly in scenarios requiring high precision and speed.

GAN also played a pivotal role in overcoming data limitations in 2024. A hybrid framework combining synthetic GAN-generated images with real-world datasets can address class imbalance challenges effectively [101]. This approach achieved 94.11% accuracy on the ELPV dataset, and the custom real-world dataset and synthetic images created with different GAN architectures, such as GAN, cGAN, and WGAN-GP, demonstrate the power of data augmentation in improving model generalization and robustness. GANs’ ability to generate high-quality synthetic data has become increasingly important in scenarios where labeled datasets are limited or imbalanced.

Multi-stage defect detection systems gained attention as well, integrating multiple imaging modalities and deep learning architectures to holistically assess PV modules. For instance, a study combined EL and thermal imaging data with advanced neural architecture, achieving cross-modal accuracy improvements of 4.2% and enabling comprehensive module assessments [102]. This innovation highlights the growing recognition of multi-modal approaches in addressing limitations of single-imaging modalities like EL.

Additionally, an improved YOLOv8 model was proposed [103], integrating several innovations to enhance both detection accuracy and computational efficiency. Key improvements included the use of Reversible Column Networks (RevCol) as the backbone to preserve feature information while reducing parameters and GFLOPs, a redesigned lightweight bottleneck fused with Efficient Multi-Scale Attention (EMA) to optimize the Neck module, and the integration of the Squeeze-and-Excitation (SE) Attention in the Head module to prioritize important channel features. These enhancements achieved a 38.46% reduction in parameters and a 34.39% reduction in GFLOPs while improving the mAP@0.5:0.95 by 2.6% compared to the baseline YOLOv8 model. These results, demonstrated on the PVEL-AD dataset, underscore the model’s potential for deployment on edge devices, offering a novel approach for real-time and resource-efficient PV defect detection.

**Figure 20:**
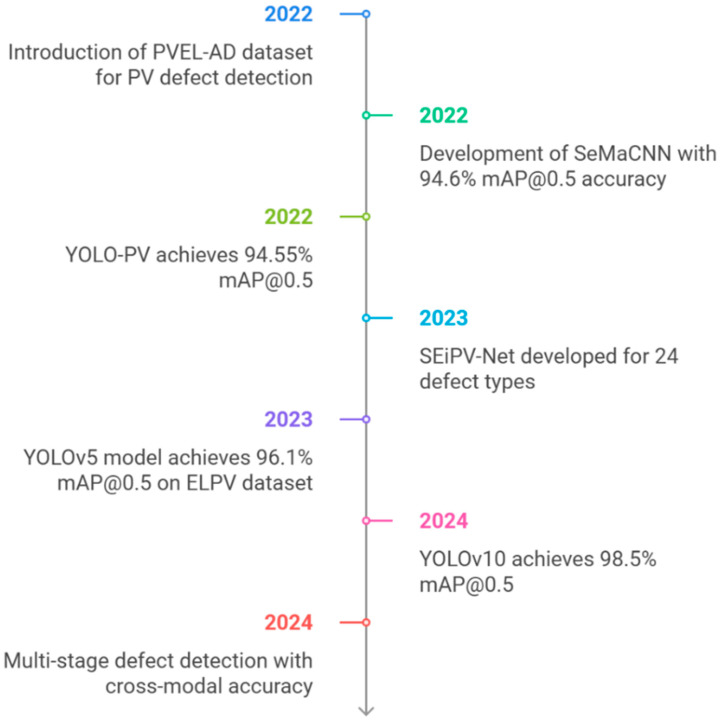
Timeline of advancements in AI-driven PV defect detection (2022–2024), highlighting key milestones such as the introduction of benchmark datasets, novel architectures, and significant performance improvements across different years.

Despite these advancements, several challenges remain. The reliance on datasets like PVEL-AD and ELPV, while beneficial for benchmarking, limits the diversity of imaging conditions represented in model training. Furthermore, while lightweight architectures and YOLO-based models demonstrated impressive performance, their robustness across different defect types, imaging modalities, and real-world conditions still needs further exploration. Similarly, the application of GANs and wavelet analysis, although promising, is yet to be fully integrated into multi-stage defect detection pipelines for holistic PV module assessment. These advancements and challenges are summarized in Figure 20, which provides a timeline of key milestones in AI-driven PV defect detection from 2022 to 2024, highlighting the evolution of datasets, models, and methodologies as the field progresses toward more scalable and robust solutions.

## 5. Future Perspectives and Research Directions

### 5.1. Challenges in Scaling Outdoor Daylight EL Imaging

The popularization of outdoor and daylight EL imaging introduces new challenges and research opportunities in the field. Daylight EL imaging faces hardware-related challenges, particularly in the acquisition system. For example, the low resolution of short-wave infrared SWIR cameras poses difficulties in developing and implementing accurate AI models that are not robust to low-resolution images.

Newer generations of PV modules tend to be larger and more powerful [109]. While their higher voltages result in stronger EL signals, they also present challenges for back-powering PV strings during in situ inspections. More powerful power supplies capable of providing sufficient voltage and current will be required to inspect entire PV strings. Additionally, back-powering becomes increasingly complex in large-scale PV plants, where entire strings must be disconnected from production to conduct EL inspections. An alternative contactless approach for in situ EL imaging is photoluminescence (PL), which utilizes sunlight as an excitation source to generate the PL signal [110]. In recent years, several methods for PL imaging have been developed, with PL signal extraction relying on switching the operational point of the modules in a string from *V_oc_* to the maximum power point (MPP). This switching can be achieved through back powering, optical modulation, string inverter operational point switching, or string IV triggering [16,62,63,65,69,111]. Even though EL and PL provide slightly different information about the nature of the defects of the cells, it has been shown that they can both be used successfully for in situ PV module characterization [112].

Traditionally, EL images have been qualitatively analyzed for defect detection. However, there is growing interest in developing quantitative models to assess degradation. Several models have been proposed to simulate power output and other IV electrical parameters using EL images as input. Empirical models utilize the relationship between luminescence and cell voltage to estimate series and shunt resistance at both the module and cell levels, which can then be used to simulate IV curves [28,113,114,115]. Alternatively, machine learning (ML) and deep learning (DL) models trained on EL images have been developed to predict power output [25,29,116,117]. However, the introduction of daylight EL imaging raises uncertainties regarding the robustness of these models. New challenges, such as increased image noise, complicate defect identification and may impact model performance. This creates an opportunity for further research, either by developing models specifically tailored for daylight EL imaging or by adapting existing models to improve their resilience to image noise and varying illumination conditions.

### 5.2. Role of AI in Enhancing EL Imaging Techniques

Despite the advancements of the AI-driven EL object detection discussed earlier in this paper, several challenges persist, limiting the full potential of AI-driven PV defect detection. These include, but are not limited to, the following:

1. Cell-Level Focus: most studies concentrate on cell-level defect detection and segmentation, often overlooking the complexities of module-level analysis. Real-world industrial systems comprise interconnected cells within larger modules, where defects can propagate and interact in ways that cell-level models cannot capture. Developing EL module-level datasets and algorithms is essential for industrial-scale deployment, as these tools must address challenges such as inter-cell electrical interactions, shading effects, and module-wide fault patterns.
2. Dataset Diversity: current datasets, such as PVEL-AD and ELPV, while foundational to the field, fail to encompass the wide variability of PV module designs and configurations. For instance, there is a lack of representation of diverse PV cell layouts, including monocrystalline, polycrystalline, full cells, and half-cut cells, which are increasingly common in modern PV systems. Additionally, module-level EL imaging datasets are severely underrepresented, limiting the ability of AI models to generalize effectively from cell-level to module-level defect detection. Furthermore, the datasets currently available do not include bifacial PV panels (cell- or module-level EL data), which are considered the next generation of PV panels, being increasingly installed in the field. This lack of diversity in dataset types restricts the adaptability and reliability of AI-driven defect detection models in addressing real-world scenarios and evolving PV technologies.
3. Single-Modality Approaches: the dominance of EL imaging limits the scope of defect detection. Non-electrical defects, such as discoloration or soiling, remain challenging to identify without incorporating visual or thermal imaging.
4. Scalability of Lightweight Models: lightweight architectures have shown promise in resource-constrained environments, offering low computational overhead. However, their scalability remains questionable when applied to complex defects or large-scale industrial PV systems. Current models often lack the robustness needed to handle diverse defect types, varying imaging modalities, or the data volume associated with operational plants, posing a barrier to their widespread deployment.
5. Integration into Industrial Pipelines: transitioning from laboratory-scale studies to industrial applications presents significant challenges. Many AI models lack real-world validation and fail to account for the complexities of operational PV plants, such as varying lighting conditions, module orientations, and degradation over time. The absence of standardized metrics and protocols for industrial-scale validation further hinders their adoption.

## 6. Conclusions

EL imaging has evolved into a cornerstone diagnostic tool in PV system assessment, playing a critical role in quality control, defect identification, and long-term performance monitoring. This review has provided a comprehensive exploration of EL imaging, tracing its progression from controlled indoor environments to the more challenging domain of outdoor daylight imaging. Through advancements in hardware, imaging techniques, and AI-driven analysis, EL imaging has significantly expanded its applicability and effectiveness in the PV industry.

The transition from traditional indoor EL imaging to outdoor and daylight EL imaging has been driven by the increasing need for rapid, large-scale PV module assessment in real-world conditions. While indoor EL imaging offers highly controlled settings for precise defect analysis, its applicability is limited by logistical challenges associated with large-scale PV installations. Conversely, outdoor EL imaging presents numerous challenges, including ambient light interference, environmental fluctuations, and operational constraints. However, significant technological advancements, particularly in infrared-sensitive InGaAs cameras, optical filtering techniques, and modulation-based signal extraction, have enhanced the feasibility of outdoor and daylight EL imaging. These innovations have mitigated noise-related challenges and improved the signal-to-noise ratio, enabling the detection of critical defects even in challenging daylight conditions.

A key breakthrough in daylight EL imaging has been the integration of periodic current modulation, which enables the differentiation of EL emissions from background noise. This method, combined with advanced image processing techniques such as lock-in amplification and Fourier transform filtering, has significantly enhanced defect detection capabilities. Additionally, drone-based EL imaging has emerged as a transformative approach, facilitating large-scale PV inspections while reducing human intervention and operational costs. Despite its promise, drone-based EL imaging still faces challenges related to motion blur, alignment accuracy, and real-time data processing, necessitating further refinement.

Artificial intelligence has revolutionized the analysis of EL images, with deep learning models demonstrating unprecedented accuracy in defect detection and classification. AI-driven methodologies have facilitated the automated segmentation of microcracks, PID, and other structural defects, surpassing traditional manual inspection techniques in both speed and accuracy. The adoption of CNNs, Transformer-based architecture, and GANs has significantly improved defect classification, anomaly detection, and performance prediction. However, challenges remain in dataset diversity, scalability, and generalization across different PV technologies and imaging conditions. Future research should focus on expanding dataset variability to encompass different module architectures, environmental conditions, and degradation modes to enhance model robustness and applicability.

While EL imaging has primarily been employed for qualitative defect detection, there is a growing interest in quantitative modeling for performance prediction. Empirical models leveraging EL image data to estimate series and shunt resistance, along with AI-driven IV curve simulations, offer promising pathways for correlating defect severity with power loss metrics. However, the transition to daylight EL imaging raises new questions about the reliability of existing models, as increased noise and environmental variability may impact defect characterization. Further studies are needed to refine these models, ensuring their applicability in real-world PV system diagnostics.

Future research directions should also explore the integration of EL imaging with complementary diagnostic techniques, such as PL and thermal imaging, to develop holistic PV health assessment frameworks. Multi-modal approaches have the potential to provide deeper insights into degradation mechanisms, module longevity, and maintenance strategies. Additionally, advancements in real-time image processing, edge computing, and AI deployment on embedded systems will be crucial in making EL imaging more accessible for large-scale industrial applications.

In conclusion, EL imaging continues to be a pivotal tool in PV diagnostics, with ongoing advancements pushing the boundaries of its applicability. The convergence of hardware innovations, AI-driven analytics, and multi-modal diagnostics is poised to further enhance its role in ensuring the reliability, efficiency, and sustainability of solar energy systems. As the PV industry continues to expand, the continued evolution of EL imaging will be instrumental in driving the next generation of high-performance, long-lasting solar technologies.

## Figures and Tables

**Table 1:** Summary of challenges in indoor and outdoor EL imaging.

Environment	Challenge	Details
Indoor EL Imaging	Module size and handling	Large module sizes complicate positioning and handling within indoor setups.
	Electrical connections	Corrosion or wear on contact points can lead to non-uniform current injection.
	Cost of setup	Expensive setup costs for high-resolution cameras and current sources.
Outdoor Dark EL Imaging	Ambient light interference	Sunlight reduces contrast, requiring shielding or advanced processing.
	Temperature fluctuations	Temperature changes alter electrical properties, impacting luminescence output.
	Weather dependence	Rain, wind, and humidity destabilize equipment and interfere with connections.
	Logistical constraints	Portable equipment is required to inspect large-scale installations quickly.
	Signal-to-noise ratio	Weak infrared signals are easily overwhelmed by external noise sources.
Outdoor Daylight EL Imaging	High ambient light levels	Requires advanced filtering techniques or specialized sensors to isolate EL signals.
	Exposure time constraints	Shorter exposure times are needed to prevent overexposure from daylight conditions.
	Equipment limitations	Daylight-compatible EL imaging systems are less mature and more expensive.
	Image post-processing	Extensive image correction and enhancement are required to extract meaningful data.
	High image volume	Typically, between 100 and 300 image frames are required per module for a good quality daylight EL image.
Shared Challenges	Resolution limitations	High-resolution systems are resource-intensive and slow for large modules.
	Electrical safety	High current injections pose safety risks, requiring rigorous protocols.

**Table 2:** Optical filter setups reported in the literature used for daylight luminescence inspections.

Reference	Optical Filter Type	Center Wavelength/Bandwidth	Application
[62]	Band-pass	~1137 nm/25 nm	Daylight PL
[63]	Band-pass	~1137 nm/25 nm	Daylight PL
[64]	Band-pass	1150 nm/30 nm	Daylight PL
[4]	Band-pass	1160 nm/150 nm	Daylight EL
[65]	Band-pass	1125 nm/50 nm	Daylight PL
[66]	Long-pass	>1000 nm	Daylight EL and PL
[3]	Band-pass	1150 nm/50 nm	Daylight drone-based EL
[67]	Band-pass	~1135 nm/0.34 nm	Daylight PL
[68]	Band-pass	1150 nm/25 nm	Daylight drone-based EL
[69]	Long-pass	>970 nm	Daylight PL

**Table 3:** Summary of commonly used EL image datasets.

Environment	Description	Link	References
ELPV	This dataset comprises 2624 grayscale images (300 × 300 pixels) of both functional and defective solar cells, extracted from 44 different solar modules. Each image is annotated with defect probability and the type of solar module (monocrystalline or polycrystalline).	https://github.com/zae-bayern/elpv-dataset (accessed on 26 March 2025)	[106,107,108]
PVEL-AD	This dataset contains 36,543 near-infrared EL images with various internal defects and heterogeneous backgrounds. It includes one class of anomaly-free images and anomalous images across twelve different categories.	https://github.com/binyisu/PVEL-AD/tree/main (accessed on 26 March 2025)	[75]
Benchmark EL Images	This repository hosts benchmark datasets (with over 15,000 cell-level) for the multi-class semantic segmentation of EL images of silicon wafer-based solar cells, providing both labeled and unlabeled images from multiple sources.	https://github.com/TheMakiran/BenchmarkELimages (accessed on 26 March 2025)	[89]
UCF EL Defect	This dataset comprises 17,064 EL images from multicrystalline and monocrystalline aluminum back-surface and monocrystalline PERC solar cells, categorized into 10 different defect classes. It also includes a segmentation tool called “DeepLabv3”.	https://github.com/ucf-photovoltaics/UCF-EL-Defect (accessed on 26 March 2025)	[105]

## Data Availability

This manuscript is a review paper, and, as such, it does not involve the generation of original data. The information presented and discussed herein is derived from publicly available sources, including published research articles, technical reports, and other relevant literature. Some figures in this paper were generated specifically for the review; however, no additional significant data are reported herein.

## Appendix A

This appendix provides a detailed explanation of the key parameters and metrics referenced in this paper to ensure clarity and understanding.

1. Mean Average Precision (mAP). mAP@0.5: The average precision calculated at a single Intersection over Union (IoU) threshold of 0.5 (50%). It measures how well the model identifies defects while considering overlap accuracy between the predicted and ground truth bounding boxes. mAP@0.5–0.95: The average precision calculated over multiple IoU thresholds (from 0.5 to 0.95 in 0.05 increments). It provides a more comprehensive evaluation of the model’s detection performance, especially for nuanced and smaller defects. Significance: mAP is a standard metric for object detection tasks, balancing precision and recall across multiple thresholds.
2. Detection Accuracy. Definition: The ratio of correctly identified defects (true positives) to the total number of predictions. It is predominantly used in classification models where the model assigns a defect type to a given input. Significance: Provides an overview of the model’s performance in distinguishing defective versus non-defective instances.

(A1)
$$Accuracy=\frac{True\;Positives+True\;Negatives}{True\;Predictions}$$

3. Average Precision (AP). Definition: The area under the precision–recall (PR) curve for a specific class or defect type. It quantifies how well a model balances precision and recall (where R is Recall). Significance: AP is used for evaluating object detection and segmentation models, offering insights into the model’s capability to detect a particular defect.

(A2)
$$AP=\int_{0}^{1}Precision\left(R\right)dR$$

4. Recall. Definition: The ratio of correctly identified positive cases (true positives) to the total actual positives (true positives + false negatives). Significance: Recall emphasizes the model’s ability to identify all instances of a defect, even at the cost of some false positives.

(A3)
$$Recall=\frac{True\;Positives}{True\;Positives+False\;Negatives}$$

5. Precision. Definition: The ratio of correctly identified positive cases (true positives) to the total predicted positives (true positives + false positives). Significance: Precision evaluates the model’s ability to avoid false alarms, ensuring the detected defects are accurate.

(A4)
$$Precision=\frac{True\;Positives}{True\;Positives+False\;Positives}$$

6. F1-Score. Definition: The harmonic mean of Precision and Recall, providing a single metric that balances both. Significance: Particularly useful when there is an imbalance between classes or when both false positives and false negatives need to be equally minimized.

(A5)
$$F1\text{-}Score=2\times \frac{Precision\times Recall}{Precision+Recall}$$

7. Intersection over Union (IoU). Definition: A measure of overlap between the predicted bounding box and the ground truth bounding box. Significance: IoU evaluates the spatial accuracy of object detection models.

(A6)
$$IoU=\frac{Area\;of\;Overlap}{Area\;of\;Union}$$

8. Area Under the Receiver Operating Characteristic Curve (AUROC). Definition: Represents the trade-off between true positive rate (sensitivity) and false positive rate (1-specificity) across different thresholds. Significance: Higher AUROC scores indicate better model discrimination capability.
